# Accuracy of dispatch and prehospital triage performance in poisonings – A retrospective study from northern Finland

**DOI:** 10.1111/aas.14152

**Published:** 2022-10-12

**Authors:** Lauri Koskela, Lasse Raatiniemi, Ari Ehrola, Timo Kaakinen, Sanna Lahtinen, Janne Liisanantti

**Affiliations:** ^1^ Research Group of Surgery, Anesthesiology and Intensive Care Medicine Medical Research Center Oulu Oulu Finland; ^2^ Centre for prehospital emergency care Oulu University Hospital Oulu Finland; ^3^ Emergency Medical Services Northern Ostrobothnian Hospital District Oulu Finland; ^4^ Department of Anesthesiology Oulu University Hospital Oulu Finland

**Keywords:** ambulances, poisoning, triage

## Abstract

**Background:**

Increasing numbers of dispatches place a burden on EMS; this study sought to assess the prehospital evaluation of poisoned patients transported to hospital. The primary aim of this study was to measure dispatch centre and EMS provider performance as well as factors contributing to the recognition of poisoning among prehospital patients. The secondary aim was to compare triage performance between dispatch centres and EMS providers.

**Methods:**

A retrospective single‐centre study in Northern Finland was conducted. Patients suspected as poisonings by dispatch centres as well as other EMS‐transported patients who received a diagnosis of poisoning in hospital between June 1, 2015 and June 1, 2017, were included.

**Results:**

There were a total of 1668 poisoning‐related EMS missions. Dispatch centres suspected poisonings with sensitivity of 79.9% (95% CI 76.7–82.9) and specificity of 98.9% (95% CI 98.9–99.0) when all EMS missions were taken into account. In a logistic regression model, decreased state of consciousness as dispatch code (OR 7.18, 95% CI 1.90–27.05) and intravenous fluid resuscitation (OR 6.58, 95% CI 1.34–32.37) were associated with EMS transport providers not recognizing poisoning. Overtriage rate appeared significantly higher (33.6%, 95% CI 28.6–39.2) for dispatch when compared with transport (17.8%, 95% CI 13.9–22.6).

**Conclusion:**

Dispatch centres seem to suspect poisonings fairly accurately. Poisonings unrecognized by EMS providers may be linked with intravenous fluid resuscitation and decreased patient consciousness. Overtriage appears to resolve somewhat from dispatch to transport. There were no fatal poisonings in this study population.

**Editorial Comment**

Dispatch centres appear to be fairly accurate in identifying poisoning as the likely main medical problem of EMS‐attended patients. The EMS teams appear to improve this further, but the final diagnosis is difficult in settings of an unclear uncosciousness of pre‐hospital patients.

## 1 INTRODUCTION

Poisonings are a heterogenic phenomenon and a major cause of accidental or suicidal death in the working‐age population. The true burden of poisonings is difficult to estimate due to variation in the categorizing of poisonings. In 2019, accidental poisonings were estimated to cause over 77,000 deaths and to account for over 4,000,000 disability‐adjusted life‐years lost globally.^1^ In Finland, the number of fatal poisonings has declined overall during the last decade, accounting for 734 deaths in 2017. Most notable has been the decline of fatal poisonings due to alcohol, with fatal poisonings by pharmaceutical and illicit drugs outnumbering fatal alcohol poisonings after 2005.^2^

The number of poisonings evaluated in healthcare units is multiple times higher than the number of fatalities. For example, Lund et al. (2012) presented data where for every poisoning death, ca. 100 poisoning patients were hospitalized and 200 evaluated as outpatients.^3^, ^4^ In a previous study done in Northern Finland, the majority of patients with fatal poisoning were found dead on scene before emergency medical services (EMS) arrival.^5^

In recent years, the increasing number of ambulance dispatches has put a burden on EMS.^6^ For acute poisoning patients, ambulance transportation is the most prevalent mode of transportation to hospital evaluation.^4^ There are studies focusing on the accuracy of triage for cardiac arrest, stroke, critical illness, trauma and also unselected patients.^7^, ^8^, ^9^, ^10^, ^11^, ^12^ Some studies on triage of poisoning patients have been conducted, but none specifically concerning prehospital dispatch or transport triage.^13^, ^14^

A single‐centre retrospective study was conducted to assess the accuracy of poisoning suspicion by dispatch centres and triage performance by EMS providers. The primary aims were to measure the accuracy of dispatch centres in suspecting poisonings and to describe which prehospital factors contributed to EMS providers' not recognizing poisonings. The secondary aim was to measure and compare triage performance between dispatch centres and EMS providers.

## 2 METHODS

### 2.1 Study design and time period

This is a retrospective study of EMS‐transported patients treated at Oulu University Hospital in Oulu‐Koillismaa Rescue Department's catchment area (population in 2017: 288,556) in Northern Finland.^15^ Oulu University Hospital is the only major hospital in the study area. Due to the retrospective nature of this study, no statement from the local ethics committee was required. The hospital administration accepted the study protocol (Ref. 190/2017). The study period was June 1, 2015, to June 1, 2017.

### 2.2 Emergency response centre in Finland

Emergency calls are assessed by criteria‐based national protocols in six emergency response centres (dispatch centres), and the appropriate EMS unit is dispatched to the scene with a symptom‐specific dispatch code. Dispatchers do not have health‐care professional status. Each mission is also categorized with respect to dispatch urgency: category A indicates a suspected life‐threatening situation, B indicates another high‐risk situation and C stands for an urgent and D for a non‐urgent situation. Lights and sirens are used in A and B missions. The EMS providers use identical symptom‐specific and urgency (transport) categories when the decision to transport the patient has been taken. In priority A dispatches, the emergency critical care (ECC) team, staffed by an EMS physician and a paramedic, is typically dispatched in addition to an ambulance.

### 2.3 Emergency department and intensive care unit

All patients transported by EMS to hospital are first admitted to the emergency department (ED). At the ED, the patient is evaluated by a physician for the first time (if the ECC team was not used for the EMS mission). In this study, hospitalization is defined by transfer from the ED to a hospital ward or an ICU. Patients can be briefly monitored at the ED, but for an overnight stay they must be admitted either to a ward or to an ICU. If the patient is in critical condition or is suspected of becoming unstable, they are admitted to an ICU.

### 2.4 Study population

**FIGURE 1:**
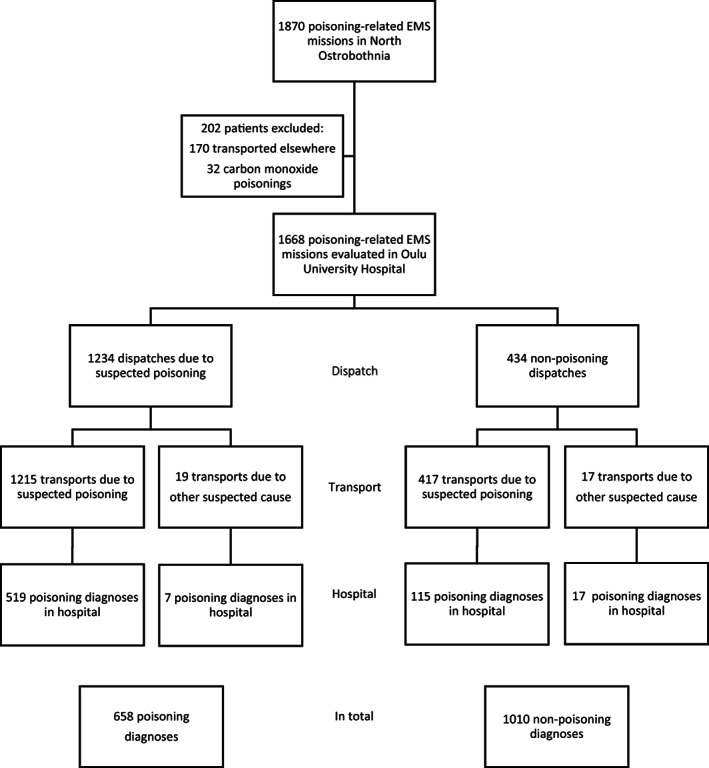
Flow chart of the study

Data were gathered on all missions where poisoning was suspected by the dispatch centre or EMS providers, based on a symptom‐specific code, and also on missions where a patient was diagnosed with poisoning in hospital but dispatch and transport were for other suspected cause. The term *poisoning‐related EMS mission* was used to include all these patients. Only patients transported to and evaluated in Oulu University Hospital were included. A minority of patients were excluded for being transported to another unit (usually a primary care centre) and for carbon monoxide poisoning. A detailed description of patient inclusion is provided in Figure 1.

### 2.5 Outcome measures

The data concerning vital parameters and suspected causes of poisonings were retrieved from the electronic EMS database (Merlot Medi). Time variables, identification number (ID) and address, dispatch and transport categories, vital signs, National Early Warning Score (NEWS, calculated from the first gathered parameters) and treatment are recorded during each EMS mission.^16^ In the electronic database, documentation is structured to perform in specific diagnosis groups such as poisonings and cardiac arrest. Patients admitted to Oulu University Hospital were identified from the hospital patient information system (Esko) discharge registry (Oberon), and data concerning hospital admission, including the need for ICU admission, discharge diagnosis and outcome, were combined with the EMS data.

### 2.6 Definitions

Patients were confirmed as poisoning patients if they were hospitalized or discharged from the ED with an ICD‐10 (International Classification of Diseases, 10th edition) diagnosis code for poisoning (X40–49, X60–69, X85–90, Y10‐Y19, T36‐T50, T51‐T65, T96, Y34, Y57, or Y84). The diagnostic code was reported by the physician based on patient history and clinical evaluation, often coupled with toxicological screening. Carbon monoxide poisonings were not included in the study population (Figure 1). If upon discharge the patient's visit had turned out to be due to a cause other than poisoning, or if the diagnostic code was missing, the admission was defined as non‐poisoning. In this study, the terms *priority dispatch category* and *priority transport category* refer to urgency categories A and B for dispatch and transport, respectively.

### 2.7 Data analysis

Statistical analysis was performed using SPSS software (IBM Corp., 2013; IBM SPSS Statistics for Windows, Version 27.0. Armonk, NY, USA). Proportional data are presented in numbers (n) and percentages (%) and continuous variables in medians and 25th–75th percentiles. Pearson's chi‐square was used to test proportional data. The distributions of continuous data were tested using the non‐parametric Mann–Whitney test. A two‐tailed *p* value <0.05 was considered statistically significant.

The accuracy of poisoning suspicion by dispatch and the success of triage for dispatch centres and EMS providers were evaluated using sensitivity, specificity, positive predictive value (PPV) and negative predictive value (NPV). For triage calculations, only patients who ended up with a poisoning diagnosis were included. Data for the number of all non‐poisoning transportations during the study period were not available, and therefore numbers reflecting the accuracy of diagnosis by EMS providers were not calculated. We defined overtriage as non‐hospitalized patients dispatched or transported with A or B urgency category. Undertriage, conversely, was defined as hospitalized patients dispatched or transported with a C or D urgency category. Overtriage rates were calculated using the established Cribari matrix method and, in addition to this, for undertriage rates a new formula proposed by Peng & Xiang was used.^17^, ^18^ The former calculates undertriaged patients in proportion to all non‐urgent missions, while the latter calculates them in proportion to all hospitalized patients. We also calculated similar rates, with the need for intensive care being determined as correct triage for the A and B mission categories.

Logistic regression analysis was used to calculate odds ratios (ORs) and 95% confidence intervals for missed poisoning diagnosis by EMS providers. All patients with a poisoning diagnosis from the hospital were included. Chosen variables entered into the model in the first step included first‐recorded age, sex, a positive alcometer finding, dichotomic NEWS variable (High risk NEWS = 7 points or more), administering 500 ml or more of intravenous (IV) fluids, priority dispatch category, altered consciousness dispatch code, distance to hospital and time spent on scene. Variables were kept in the model if the *p* value was <0.05 or if they had significant impact on the –log likelihood function. A correlation matrix was used to identify potential collinearity.

## 3 RESULTS

During the study period there were a total of 67,747 EMS missions, and in 1870 of these missions poisoning was suspected or confirmed at some point and the patient was evaluated in the ED. After excluding patients who were transported elsewhere (9.1% of missions) and carbon monoxide poisonings (1.7% of missions), there were 1668 missions remaining. In 1632 (97.8%) of these transports poisoning was suspected and in 36 (2.2%) transports another cause was suspected (Figure 1). A detailed flow chart is presented in Figure 1. In 541 (32.4%) patients the diagnosis code was missing upon discharge, and these patients were included in the non‐poisoning category.

In total, 58 (3.5%) of 1668 patients studied were admitted to the ICU. There was one fatality in the non‐poisoning population. The death was contributed to convulsion and subdural hematoma in hospital.

For 309 poisoning suspects, information on ingested agents was obtained as part of the patient history. Combined poisoning by drugs and alcohol in 91 (29.4%) cases was the most prevalent type, closely followed by single‐agent poisoning by a pharmaceutical drug in 89 (28.8%) cases. In 55 (17.8%) cases, multiple pharmaceutical drugs were suspected. Pure alcohol poisoning was reported in 42 (13.6%) cases. In 27 (8.7%) cases an illicit drug and in 5 (1.6%) cases a miscellaneous agent was involved.

### 3.1 Differences between recognized and unrecognized poisonings

Poisonings identified correctly by EMS were more likely to have a positive alcometer finding, but less likely to have disturbances in collected vital parameters than unrecognized poisonings according to NEWS. Unrecognized poisonings were also more likely to have an altered consciousness dispatch code and were administered ≥500 ml of intravenous fluids more frequently (Table 1).

**TABLE 1:** Univariate data for 658 poisoning‐related missions. Data are presented as numbers (percentages) or medians (25th‐75th percentiles). Percentages are in relation to nonmissing data

Variable	Poisonings transported as poisonings *n* = 634	Poisonings transported as another suspected cause *n* = 24	*p* value
Male	367 (58.3)	15 (62.5)	0.834
Age	39.1 (28.1–49.7)	42.4 (31.9–55.1)	0.164
Priority dispatch category	441 (69.6)	20 (83.3)	0.177
Poisoning dispatch code	519 (81.9)	7 (29.2)	<0.001
Altered consciousness dispatch code	10 (1.6)	9 (37.5)	<0.001
Positive alcometer	331 (52.2)	5 (20.8)	.003
High‐risk NEWS (≥7)	251 (39.6)	19 (79.2)	<0.001
Fluid resuscitation ≥500 ml	294 (46.4)	18 (75.0)	<0.001
Distance from scene to hospital, km	5.5 (3.9–9.4)	8.0 (4.1–33.2)	0.183
Time spent on scene, min	19 (13–26)	23 (12.5–49.5)	0.214

Abbreviation: NEWS, national early warning score.

In the logistic regression model, the findings of the univariate analysis in Table 1 persisted (Table 2). The performance of the model was significantly impacted by a lack of events (ca. 1.3 events per variable), and while the omnibus *p* value for model remained <0.001, the *p* value for Hosmer‐Lemeshow goodness‐of‐fit rose to 0.983.

**TABLE 2:** Odds ratios (OR) and 95% confidence intervals (CI) for factors associated with unrecognized poisonings by EMS providers during prehospital care according to logistic regression analysis

Variable	Unadjusted OR (95% CI)	OR (95% CI)	*p* value
Decreased consciousness as dispatch code	37.4 (13.3–105.5)	7.18 (1.90–27.05)	0.004
Poisoning dispatch code	0.091 (0.037–0.225)	0.057 (0.011–0.29)	<0.001
Fluid resuscitation ≥500 ml	3.47 (1.36–8.86)	6.58 (1.34–32.37)	0.021
Positive alcometer	0.24 (0.089–0.65)	0.096 (0.012–0.78)	0.029

### 3.2 Accuracy of poisoning suspicion and triage

The poisoning mission code upon dispatch had a sensitivity of 79.9% (95% CI 76.7–82.9) and specificity of 98.9% (95% CI 98.9–99.0) (Table 3). For the triage of priority dispatch, the overtriage rate was 33.6% (95% CI 28.6–39.2). For priority transport, the corresponding overtriage rate was 17.8% (95% CI 13.9–22.6). Hospital admission was assumed as an appropriate triage for a priority mission (Table 4). Alternatively, when the need for ICU admission was assumed to be appropriate triage, the respective overtriage rates were 89.4% (95% CI 81.1–98.3) for priority dispatch and 86.3% (95% CI 77.2–96.2) for priority transport. Also, only 49 (10.6%) of the priority dispatch patients and 50 (13.7%) of the priority transport patients were admitted to an ICU.

**TABLE 3:** Confusion matrix for accuracy of poisoning suspicion by dispatch centres. Proportions are presented as percentages (95% confidence interval)

	Poisoning dispatch	Other dispatch	Total
Poisoning	526	132	658
Non‐poisoning	708	66,381	67,089
Total	1234	66,513	67,747
Sensitivity	79.9 (76.7–82.9)		
Specificity	98.9 (98.9–99.0)		
PPV	42.6 (40.6–44.7)		
NPV	99.8 (99.8–99.8)		

Abbreviations: NPV, negative predictive value; PPV, positive predictive value.

**TABLE 4:** Confusion matrix for triage in dispatch and transport for poisoning patients when the need for hospital admission was assumed as an appropriate triaging for priority dispatch. Proportions are presented as percentages (95% confidence interval)

	Priority dispatch	Normal dispatch	Priority transport	Normal transport	Total
Hospital admission	306	85	300	91	391
Discharge from ED	155	112	65	202	267
Total	461	197	365	293	658
	Dispatch		Transport		
Sensitivity	78.3 (73.8–82.3)		76.7 (72.2–80.8)		
Specificity	42.0 (36.0–48.1)		75.7 (70.1–80.7)		
PPV	66.4 (63.8–68.9)		82.2 (78.8–85.2)		
NPV	56.9 (51.0–62.5)		68.9 (64.7–72.9)		
Overtriage	33.6 (28.6–39.2)		17.8 (13.9–22.6)		
Undertriage	43.1 (34.7–53.1)	21.7^a^ (17.5‐26.8)	31.1 (25.2–38.0)	23.3^a^ (18.9‐28.4)	

Abbreviations: ED, emergency department; NPV, negative predictive value; PPV, positive predictive value.

^a^ Undertriage rate calculated using the Peng and Xiang method.

## 4 DISCUSSION

This study revealed that dispatch centres are correct in poisoning suspicion most of the time, with 79.9% sensitivity and 98.9% specificity when all dispatches are taken into account. The overtriage rate decreased from 33.6% to 17.8% from dispatch to transport. Unrecognized poisoning patients appear to have disturbances in vital parameters, and interventions such as intubation and fluid resuscitation are more common. A dispatch code of decreased consciousness and need for fluid resuscitation were significant factors contributing to failed recognition of poisoning patients by EMS providers.

### 4.1 Accuracy of EMS diagnosis and triage

The dispatch centres' accuracy of poisoning suspicion seemed high in these data. Some studies concerning the accuracy of suspected cause of prehospital care were found in the literature, at least for out‐of‐hospital cardiac arrests, prehospital stroke diagnosis and trauma.^19^, ^20^, ^21^, ^22^, ^23^ No similar studies on the accuracy of poisoning suspicion were found.

For hospital admission we found a dispatch overtriage rate of 33.6%. The overtriage rate was 89.4% when ICU admission was determined as the correct priority mission triage. Even though it has been previously suggested that the increase in EMS missions is due to patients requesting ambulances for less severe illnesses and injuries than those in the study population, this result still suggests that EMS resources (especially more critical units) could be spared by reducing overtriage.^6^ It is, however, challenging to determine a correct corresponding in‐hospital outcome with which to assess the triage of priority EMS missions. In this study, we decided to choose hospitalization as the main corresponding triage but also calculated values for ICU admission, as A and B priority missions are supposedly reserved for life‐threatening situations. As can be seen from the results however, only a few patients (10.6% of priority dispatches, 13.7% of all poisoning patients) ended up in the ICU. In previous studies, triage performance has been compared against the NACA score (National Advisory Committee for Aeronautics, score predicts prehospital severity of patient)^24^, ^25^ and a combination of injury severity score (ISS), emergency intervention, ICU admission or death,^22^ for example. Also, as the findings from the univariate analysis might suggest, patients with unrecognized poisonings (even more so than other poisonings) often present with transient disturbances in vital functions, judging from the drastically increased percentage of high‐risk NEWS patients among them (Table 1). Hence, overtriaging is mandatory upon dispatch when the triage decision is made with limited information. If the patient could be suffering from another condition leading to a decreased state of consciousness, rather than a poisoning with a usually excellent outcome, swift action is warranted. One encouraging result in this study was that the overtriage rate decreased significantly from dispatch to transport, when compared against either hospital or ICU admission. For hospital admission the decrease was, remarkably, from 33.6% to 17.8%. This could be due to overtriage on dispatch, improvement of the patient's condition upon EMS arrival, the result of EMS intervention or some unknown cause.

An interesting result was that there appeared to be an increase in the undertriage rate from dispatch to transport (from 21.7 to 23.3) when it was calculated using the method recommended by Peng and Xiang, and a decrease (from 43.1 to 31.1) when the original Cribari Matrix method was used.^18^, ^26^ In the former method all hospitalized patients are included in the denominator, while the latter uses all non‐urgent missions as the denominator. And while the number of hospitalized patients remained similar for both dispatch and transport, the number of non‐urgent transports was notably greater than the number of non‐urgent dispatches. This should explain the discrepancy (Table 4). Also, the former result did not appear to be statistically significant.

### 4.2 Differences between recognized and unrecognized poisonings

Poisonings unrecognized by EMS providers had lower consciousness dispatch codes more often than other poisonings (Tables 1 and 2). High OR (7.18, 95% CI 1.90–27.05) for decreased consciousness as a symptom‐specific dispatch code might suggest that these poisoning patients were first treated more generally as loss‐of‐consciousness patients. In a majority of cases, other reasons for decreased consciousness, such as stroke, require quicker interventions than poisonings to achieve good outcome. Therefore, mistaking poisoning patients as decreased‐consciousness patients seems acceptable. Interestingly, unrecognized poisoning patients appeared to require fluid resuscitation more often than patients who were correctly transported as poisonings. This could be linked to the decreased consciousness of unrecognized poisoning patients: patients with decreased consciousness are usually administrated intravenous fluids as a normal EMS protocol. The same might not always be true when treating a suspected poisoning with normal vital parameters and good clinical condition, but this explanation remains speculative. This explanation would also suggest significant collinearity between decreased consciousness and fluid resuscitation, which was not present in the model. Another explanation is that the overall condition of these patients was more severe, perhaps including hypotension, hypovolemia or another reason that caused paramedics to administer a significant amount of intravenous fluid. Also, a paramedic's reasoning regarding need for intravenous cannulation and often subsequent fluid resuscitation is affected by multiple different factors; for example, disturbance in different vital functions, administration of medicine or analgesia, need for blood samples, etc.^27^ At best, our result concerning fluid resuscitation can be interpreted as a preliminary finding.

That positive alcometer findings contributed to recognized poisonings is an expected result. Even though data on poisoning substances were not gathered in this study, the prior literature allows us to hypothesize that a significant number of alcohol poisonings are present in this population.^28^, ^29^

### 4.3 Mortality

There were no fatal poisonings in this study population. A low in‐hospital mortality rate for poisonings has already been demonstrated in the literature.^30^, ^31^, ^32^, ^33^, ^34^ This again supports the prospect that, in terms of outcome, there is only little to be gained by improving the management of poisonings in the pre‐hospital or in‐hospital setting and that resources might be diverted to other patient groups with more urgent needs.

### 4.4 Limitations

The study population included a large number of patients for whom there was no diagnosis code (32.4%). For these patients, other data were recorded normally. Local hospital guidelines make ICD‐10 reporting mandatory for each visit, with a physician's evaluation, but this is not strictly enforced and a missing diagnosis code can be forgotten by the physician. This may have left potential poisoning patients outside the poisoning patient group, and the results of this study should thus be interpreted with caution. However, this phenomenon will occur at random and we believe that it should not affect the results significantly. The accuracy of poisoning diagnosis by a physician is also susceptible to human error; however, as poisonings represent only a minority of emergency patients, poisoning diagnoses are usually based on either a positive screening result or strong suspicion of intake. Although not a gold standard, hospital‐evaluated poisoning diagnosis remains the best practical parameter for this study population.

The accuracy of poisoning suspicion in this study is interpreted from the symptom‐specific code, and if two or more valid suggested codes are plausible, only one can be reported. If poisoning was the excluded option, our study cannot take this into account. But as mentioned earlier for physicians, poisoning suspicion in dispatch is usually based on strong suspicion of intake and in the above‐mentioned cases it is more likely to be reported.

Poisoning agents were also not reported, as they are known with certainty in only a minority of patients for whom a screening test is conducted. Moreover, multiple poisonous substances are often present.

Potential out‐of‐hospital poisoning deaths may have been excluded due to the study's exclusion criteria. In previous work covering same study area, a majority of poisoning fatalities were found dead before EMS arrival.^5^ Therefore, the number of poisoning fatalities on scene or during transport should be limited. From the EMS protocol, we know that patients transported to a health centre are supposedly those with stable vital functions and therefore should have even better outcomes than poisonings admitted to a hospital. The health centre–treated population should then not be at risk for severe sequelae if mistreated by the EMS.

The logistic regression model ended up being statistically weak due to the low number of reference events (poisonings transported as non‐poisonings; Figure 1). Nevertheless, the model upheld the findings from univariate analysis.

## 5 CONCLUSION

In this population of poisoning‐related EMS missions, dispatch centres appear to suspect poisonings fairly accurately. Overtriage for dispatch seems to resolve somewhat upon transport. Unrecognized poisonings by EMS providers might be linked with decreased consciousness upon dispatch and need for fluid resuscitation. There were no fatal poisonings in this study, further confirming the good outcomes in the hospital‐treated poisoning population.

## AUTHOR CONTRIBUTIONS

LK, LR, AE and JL designed the study, collected and analysed the data. LK, LR, AE, TK, SL and JL all collaborated in writing the manuscript.

## FUNDING INFORMATION

This study received funding from the Finnish Foundation for Alcohol Studies and the Finnish Medical Society Duodecim. LK has received a grant (Eka/2019) from The Finnish Medical Society.

## CONFLICT OF INTEREST

Authors declare no conflict of interest.
